# Data on phenylalanine-to-tyrosine ratios in assessment of tetrahydrobiopterin (BH_4_)-responsiveness in patients with hyperphenylalaninemia

**DOI:** 10.1016/j.dib.2022.107926

**Published:** 2022-02-04

**Authors:** Barbka Repic Lampret, Mojca Zerjav Tansek, Blaz Groselj, Jaka Sikonja, Tadej Battelino, Urh Groselj

**Affiliations:** a Clinical Institute for Special Laboratory Diagnostics, University Children's Hospital, UMC Ljubljana, Ljubljana 1000, Slovenia; b Department of Pediatric Endocrinology, Diabetes and Metabolic Diseases, University Children's Hospital, UMC Ljubljana, Ljubljana 1000, Slovenia; c Faculty of Medicine, University of Ljubljana, Ljubljana 1000, Slovenia; d Institute of Oncology Ljubljana, Ljubljana 1000, Slovenia

**Keywords:** Phenylketonuria, Phenylalanine-to-tyrosine ratio, Tetrahydrobiopterin, BH_4_-responsiveness, Hyperphenylalaninemia, Tandem mass spectrometry, BH_4_, tetrahydrobiopterin, cPKU, classic phenylketonuria, F, female, M, male, MHP, mild hyperphenylalaninemia, mPKU, mild phenylketonuria, NI, not included, NR, non-responder, Phe, phenylalanine, Phe/Tyr-ratio, phenylalanine-to-tyrosine ratio, R, responder, Tyr, tyrosine

## Abstract

Blood phenylalanine-to-tyrosine (Phe/Tyr) ratio is an important indicator of metabolic control in phenylketonuria patients. We present the data that highlights the role of Phe/Tyr-ratio in the evaluation of tetrahydrobiopterin (BH_4_)-responsiveness in patients with hyperphenylalaninemia. Our data complements the results from the original research article by Tansek et al., 2012 [1]. We performed a BH_4_-loading test in 32 patients after four days of increased protein intake (2000 mg/kg body weight). Blood sampling was performed 96, 72, 48, 24, 16 h, and moments before oral administration of BH_4_ in a dose of 20 mg/kg body weight. Additional blood samples were collected 8 and 24 h after its administration. Phenylalanine (Phe) and Tyrosine (Tyr) levels were determined from dried blood spots by tandem mass spectrometry. Phe/Tyr-ratio reached a plateau after three days of increased dietary protein intake. Fifteen patients (47%) responded to BH_4_, defined as a decrease of Phe-of at least 30% after 24 h of BH_4_ administration. Phe/Tyr-ratios were significantly higher in non-responders compared to responders. In the responder group, Phe/Tyr-ratios decreased in average of 67% (*p* = 0.001) and 45% (p = 0.001) after 8 and 24 h of BH_4_ administration, respectively. Phe/Tyr-ratio decreased after 8 h of drug administration also in the non-responder group, but not 24 h after administration.

## Graphical abstract

**Figure:**
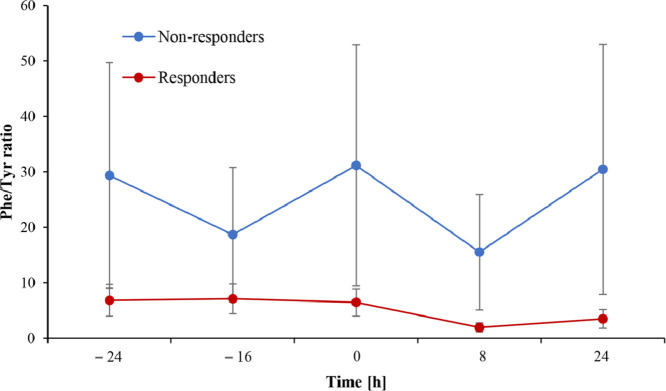

## Specifications Table

**Table:** 

Subject	Endocrinology, Diabetes and Metabolism
Specific subject area	Inborn errors of metabolism; Hyperphenylalaninemia; Phenylketonuria; tetrahydrobiopterin responsiveness
Type of data	Table Chart Figure
How the data were acquired	Dried blood spots were analysed by Tandem Mass Spectrometry. All data was acquired from electronic medical records and analysed by Microsoft Office 365 Excel and IBM SPSS Statistics, version 26.0.
Data format	Raw
Description of data collection	To assess the response to tetrahydrobiopterin (BH_4_), patients ceased with their nutritional supplements for hyperphenylalaninemia and started receiving a protein intake of 2000 mg/kg body weight for five days. After four days on a modified diet, they received 20 mg/kg body weight of BH_4_. Following acquisition, blood samples were smeared on a special filter paper.
Data source location	UMC - University Children's Hospital Ljubljana Ljubljana Slovenia 3G3C + MM Ljubljana, Slovenia
Data accessibility	Repository name: Mendeley Data DOI: 10.17632/hr7y3h7dsz.1 Direct URL to data: https://data.mendeley.com/datasets/hr7y3h7dsz/1
Related research article	M.Z. Tansek, U. Groselj, S. Murko, H. Kobe, B.R. Lampret, T. Battelino, Assessment of tetrahydrobiopterin (BH(4))-responsiveness and spontaneous phenylalanine reduction in a phenylalanine hydroxylase deficiency population, Mol. Genet. Metab. 107(1-2) (2012) 37-42. doi: 10.1016/j.ymgme.2012.07.010.

## Value of the Data

• The data supports the use of phenylalanine-to-tyrosine (Phe/Tyr) ratio in the assessment of tetrahydrobiopterin (BH_4_)-responsiveness.
• The data could help physicians in predicting the response to BH_4_ in patients with hyperphenylalaninemia.
• The data could be used by field experts for the development of personalized treatments for patients with hyperphenylalaninemia.
• Further studies of BH_4_-responsiveness could benefit from the addition of Phe/Tyr-ratio in metabolic monitoring.
• Due to the rarity of hyperphenylalaninemia-causing errors of metabolism, additional data on treatment selection is beneficial.

## 1 Data Description

### 1.1 Patients’ characteristics

We included 32 patients (17 females) with a median age of 5.7 years. The youngest patients was 1 year old and the oldest 22 years. According to BH_4_-responsiveness criteria, 15 patients were responders (9 females) and 17 non-responders (8 females). Age difference between the two groups was not statistically significant (*p* = 0.355), although metabolic phenotypes were differently distributed between the responder and non-responder groups. Nine patients with classic phenylketonuria (cPKU), 4 with mild phenylketonuria (mPKU) and 4 with mild hyperphenylalaninemia (MHP) were in the non-responder group, while in the responder group none had cPKU, 8 had mPKU and 7 MHP. No side effects were observed during the BH_4_-loading test. Detailed patients’ characteristics are presented in Table 1.

**Table 1:** Patient characteristics and tetrahydrobiopterin loading results.

#^a^	#^b^	Gender	Age [year]	Metabolic Phenotype^c^	BH_4_-resp.^d^	Phe-red. 24 h [%]^e^	Phe/Tyr 0 h	Phe/Tyr 8 h	Phe/Tyr 24 h	Phe/Tyr-red. 8 h [%]	Phe/Tyr-red. 24 h [%]
1	27	M	8	cPKU	NR	6.9	56.90	30.51	51.90	46.4	8.8
2	33	F	7	cPKU	NR	-1.5	44.35	21.82	40.94	50.8	7.7
3	34	F	2	cPKU	NR	-7.3	43.82	18.42	36.12	58.0	17.6
4	30	M	4	MHP	NR	-14.3	6.04	4.34	4.78	28.1	20.8
5	15	M	1	cPKU	NR	9.9	37.90	21.79	37.24	42.5	1.7
6	17	F	2	MHP	NR	-10.6	7.10	6.01	7.03	15.4	1.0
7	23	F	3	cPKU	NR	-15.6	36.01	18.80	33.21	47.8	7.8
8	28	M	7	cPKU	NR	12.4	61.04	28.69	69.96	53.0	-14.6
9	35	M	3	cPKU	NR	15.6	56.81	25.54	61.95	55.0	-9.0
10	26	F	8	cPKU	NR	-9.5	55.13	22.64	56.30	58.9	-2.1
11*	16	M	2	cPKU	NR	-1.9	44.48	24.84	37.57	44.2	15.5
12*	20	F	18	mPKU	NR	-12.7	27.76	10.47	29.17	62.3	-5.1
13*	NI	M	6	mPKU	NR	9.9	3.21	0.68	3.82	78.8	-18.8
14*	36	F	15	mPKU	NR	-13.6	36.46	21.51	38.10	41.0	-4.5
15	11	M	4	MHP	NR	27.6	2.51	0.79	1.56	68.7	37.7
16	13	M	4	MHP	NR	29.1	5.15	4.37	4.40	15.2	14.6
17*	19	F	12	mPKU	NR	22.2	5.29	2.86	3.84	45.9	27.3
18	9	M	8	mPKU	R	66.8	4.71	1.03	2.24	78.1	52.5
19	21	F	4	MHP	R	67.9	5.79	2.12	1.57	63.4	72.9
20	7	M	2	mPKU	R	48.7	8.50	2.40	5.37	71.7	36.9
21	10	F	8	MHP	R	72.5	3.38	1.03	1.40	69.6	58.5
22	3	F	17	mPKU	R	43.0	9.93	2.60	6.11	73.8	38.5
23	4	F	14	mPKU	R	54.3	8.64	1.90	4.51	77.9	47.8
24	NI	M	4	MHP	R	37.1	1.84	1.10	1.52	40.1	17.3
25	NI	F	2	MHP	R	30.7	7.13	3.04	6.05	57.4	15.2
26	12	F	2	mPKU	R	46.6	6.84	1.09	3.33	84.1	51.3
27	2	M	5	MHP	R	45.3	6.76	2.78	4.92	58.8	27.1
28	14	F	9	MHP	R	44.5	4.15	0.59	2.94	85.8	29.0
29*	5	M	8	mPKU	R	44.8	7.90	2.83	3.87	64.2	51.0
30*	18	F	7	mPKU	R	43.1	3.99	2.88	2.23	27.7	44.0
31*	6	M	4	MHP	R	71.1	7.18	2.11	1.69	70.6	76.5
32*	8	F	22	mPKU	R	65.9	10.36	1.82	4.59	82.4	55.7

a Patient number. Patients 22 and 23, and patients 17 and 30 are siblings. * – Patients with all pre-BH_4_-loading amino acid measurements.

b Patient number from the original research article. NI – not included.

c Phenotype as determined by their pre-treatment plasma phenylalanine concentrations: cPKU – classic phenylketonuria; mPKU – mild phenylketonuria; MHP – mild hyperphenylalaninemia.

d R – responder (phenylalanine reduction ≥ 30% after 24 h of BH_4_ administration); NR – non-responder.

e Measured by tandem mass spectrometry from dried blood spots. Abbreviations: BH_4_ – tetrahydrobiopterin; F - female; M – Male; Phe – phenylalanine; Phe/Tyr - phenylalanine-to-tyrosine ratio; red. – reduction; resp. – responsiveness.

### 1.2 Tyrosine (Tyr) concentration and Phe/Tyr-ratio pre-administration of BH_4_

**Fig. 1:**
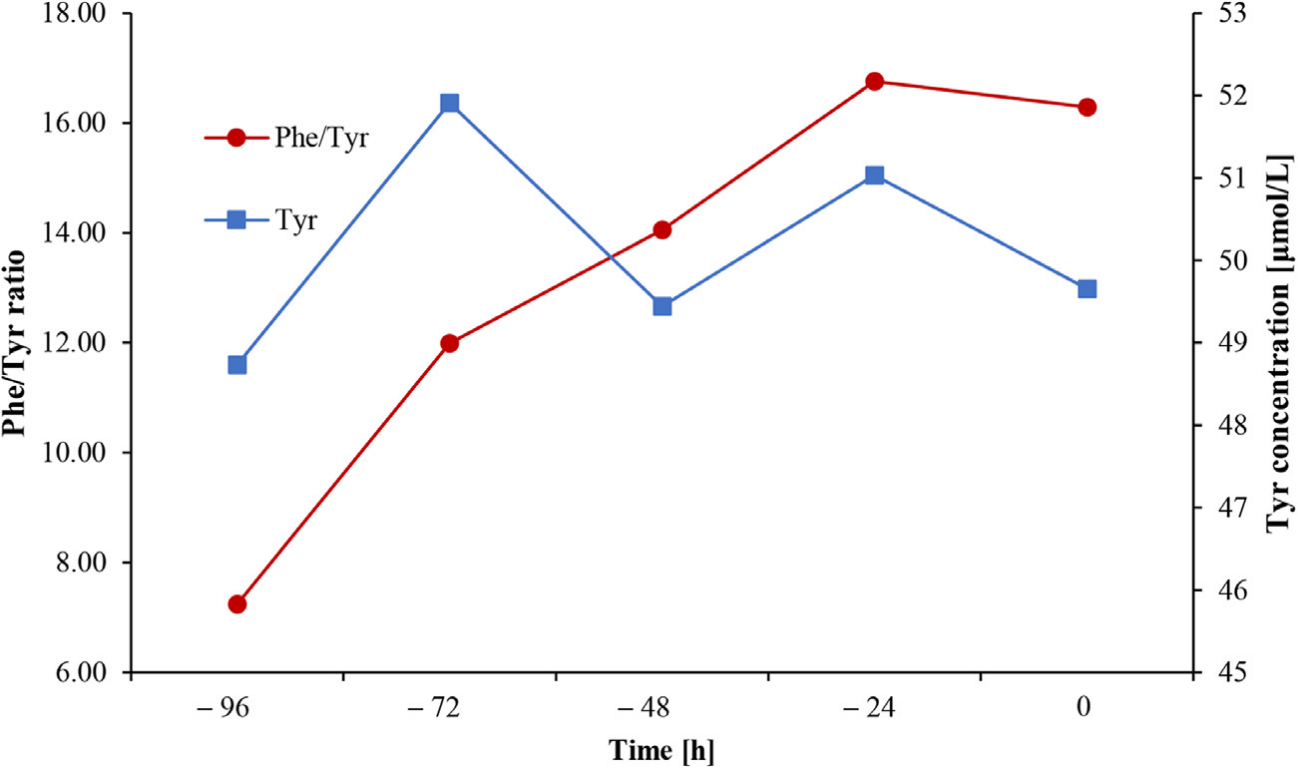
Phe/Tyr-ratios and Tyr-concentrations prior to BH_4_ administration.

We monitored blood Phe, Tyr-and Phe/Tyr-ratios in nine patients (5 females) at t_–96_, t_–72_, t_–48_, t_–24_ and t_0_. After 3 days of increased dietary intake of protein, blood phenylalanine (Phe) concentrations reached a plateau. As observed with Phe, Phe/Tyr-ratio rose steadily for 3 days and also reached a plateau at a mean of 16.53 (Fig. 1). Tyr-concentrations stayed relatively stable during the test. The average of mean Tyr-concentrations from different time points was 50.16 µmol/L (standard deviation = 1.29 µmol/L).

Phe/Tyr-ratio and Tyr-concentrations were evaluated separately for nine patients: 4 responders and 5 non-responders. The marks on the x-axis of the figure represent the time prior to BH_4_ administration and dots represent the average Tyr-concentrations (blue) and Phe/Tyr-ratios (red). As Phe/Tyr-ratio steadily rose and reached a plateau after three days of increased dietary protein uptake, Tyr-concentration remained stable. Abbreviations: Phe/Tyr-ratio – phenylalanine-to-tyrosine ratio; Tyr – tyrosine.

### 1.3 Phe/Tyr-ratios regarding to phenotype

**Fig. 2:**
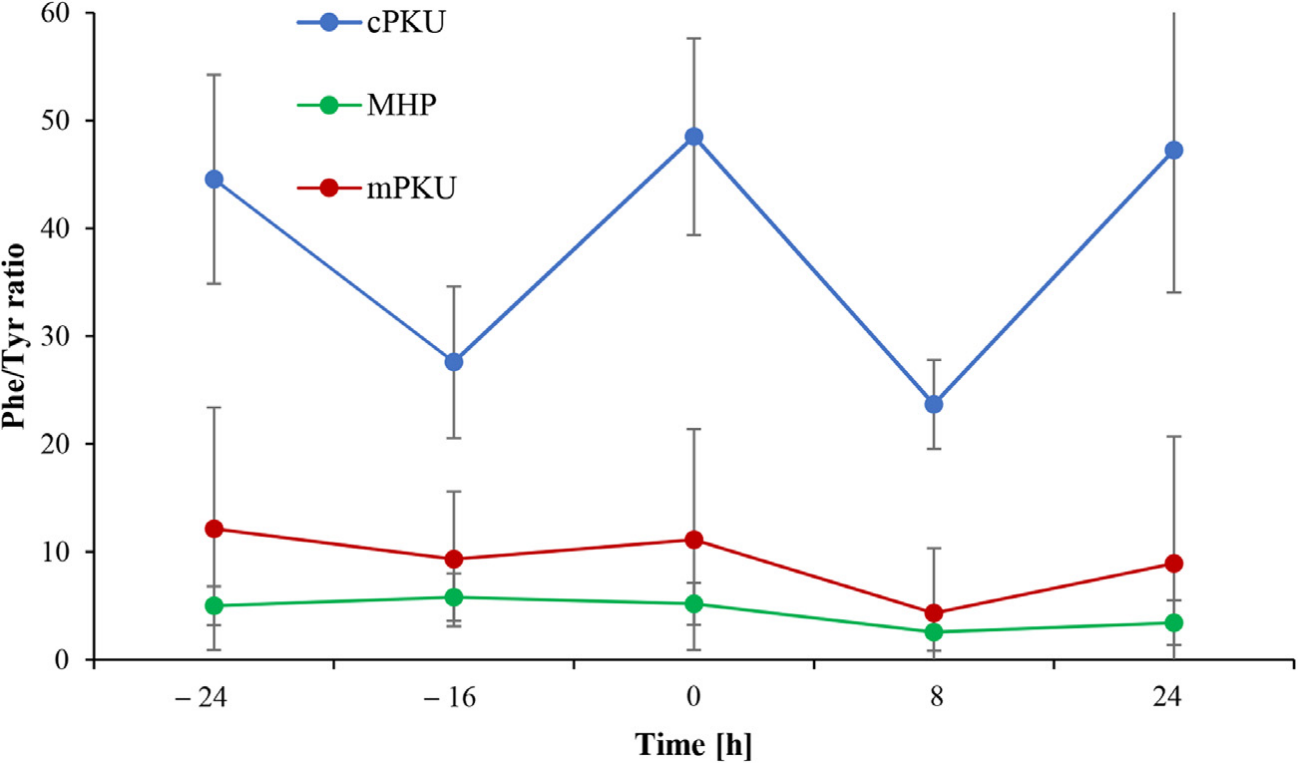
Phe/Tyr-ratios and Tyr-concentrations across different metabolic phenotypes.

We compared Phe/Tyr-ratios in patients with different metabolic phenotypes. The highest ratios were observed in cPKU patients compared to mPKU and MHP (*p* < 0.001 for both comparisons). Phe/Tyr-ratios were comparable between mPKU and MHP whereas mPKU patients had slightly higher ratios (Fig. 2). This difference was statistically significant only for Phe/Tyr-ratios at t_−24_ and t_0_ (*p* = 0.019 and p = 0.042, respectively). One day after receiving BH_4_, there was a significant fall of Phe/Tyr-ratio in both mPKU and MHP patients relative to baseline levels (t_0_), however this finding was not observed in cPKU patients (*p* = 0.012, *p* = 0.003 and *p* = 0.515, respectively). In the latter group, we can see an initial decrease of Phe/Tyr-ratios after 8 h of BH_4_ administration which was followed by an increase to baseline concentrations 24 h after loading.

The figure shows average Phe/Tyr-ratios with standard deviations in patients from three phenotype groups: cPKU (blue), mPKU (red) and MHP (green). Patients with cPKU had higher Phe/Tyr-ratios throughout the observation period compared to the other two groups. mPKU and MHP patients experienced a decrease of Phe/Tyr-ratio after 8 and 24 h from BH_4_ administration. Abbreviation: cPKU – classic phenylketonuria; MHP – mild hyperphenylalaninemia; mPKU – mild phenylketonuria; Phe/Tyr-ratio – phenylalanine-to-tyrosine ratio.

### 1.4 Phe/Tyr-ratio regarding to BH_4_-responsiveness

**Fig. 3:**
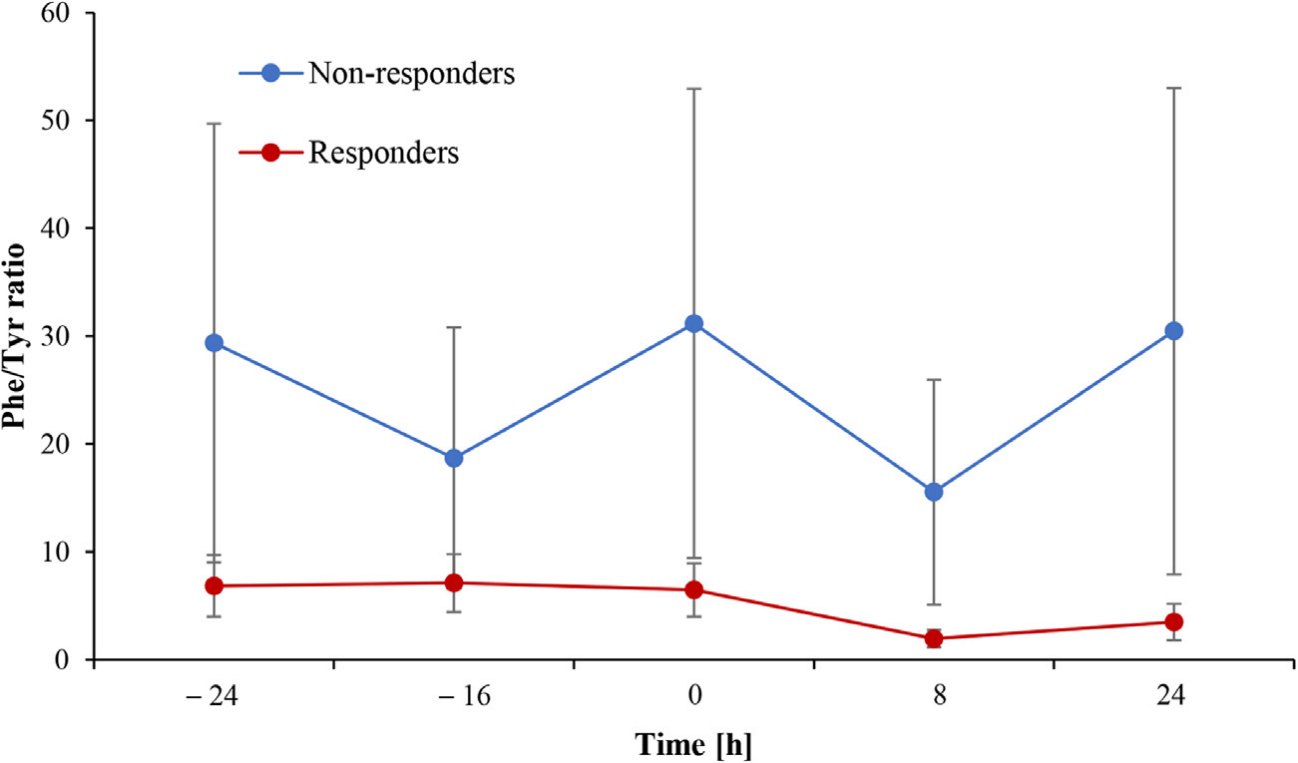
Phe/Tyr-ratios and BH_4_-responsiveness.

Phe/Tyr-ratios between BH_4_-responders and non-responders were significantly different throughout the observation period (*p* < 0.001) and are shown in Fig. 3. In the responder group, Phe/Tyr-ratio decreased in average of 67% (*p* = 0.001) and 45% (p = 0.001) from baseline concentrations after 8 and 24 h of BH_4_ administration, respectively. On the other hand, a decrease of Phe/Tyr-ratio from baseline was evident at 8 h after BH_4_-loading and not at 24 h in the non-responder group as Phe/Tyr-ratio decreased in average 48% (*p* < 0.001) and 6% (*p* = 0.356) after 8 and 24 h from BH_4_-loading, respectively.

Average Phe/Tyr-ratios together with standard deviations are presented for two groups according to the response to BH_4_. Non-responders (blue) had higher Phe/Tyr-ratios throughout the observation period compared to responders (red). Additionally, responders experienced a decrease of Phe/Tyr-ratios and had lower Phe/Tyr-ratios at 8 h and 24 h after BH_4_-administration, while in the non-responders group Phe/Tyr-ratios were lower only 8 h after drug administration. Abbreviation: Phe/Tyr-ratio – phenylalanine-to-tyrosine ratio.

Dataset title: Phenylalanine-to-tyrosine ratio in assessment of tetrahydrobiopterin responsiveness in hyperphenylalaninemia

Dataset description: Dataset includes the general patient information (gender, age) and their metabolic phenotype. Additionally, the data on Phe, Tyr-levels and Phe/Tyr-ratios at various time points is included.

## 2 Experimental Design, Materials and Methods

### 2.1 Participants of the study and collection of samples

To assess the role of Phe/Tyr-ratio in BH_4_-reponsiveness assessment, we included (i) patients with at least one mutation that was BH_4_-responsive, (ii) patients with at least one mutation with unclear effect on BH_4_-responsiveness and (iii) patients that were tested for BH_4_-responsiveness before the results of genetic analyses. The data on mutations and genotypes related BH_4_-responsiveness were obtained from BIOPKU database (available at: www.biopku.org/biopku). Genetic characteristics of the included patients were previously described in the original research article by Tansek et al. [1]. Altogether, thirty-two patients (17 females) were selected for the study, were receiving dietary treatment for hyperphenylalaninemia and were regularly followed as outpatients.

### 2.2 Metabolic phenotype

Pre-treatment plasma Phe-levels or dietary Phe-tolerance were used to classify participants into three phenotype groups: cPKU (Phe ≥ 1200 μmol/L; Phe-tolerance ≤ 350 mg/day), mPKU (Phe 600–1200 μmol/L; Phe-tolerance 400–600 mg/day) and MHP (Phe < 600 μmol/L on a normal diet) [1,2].

### 2.3 Dietary protein intake and BH_4_-loading test

Included patients stopped taking their PKU nutritional supplements for five days during the study. At the same time, each patient received her/his own individual meal plan, prepared by a dietician based on her/his food choices. Protein intake was increased to 2000 mg/kg body weight, corresponding to an approximate Phe-intake of 100 mg/kg body weight. Adequacy of dietary intake was controlled on site.

Four days after starting the study, each patient received 20 mg/kg body weight of BH_4_ (Kuvan®, sapropterin dihydrochloride, Merck Sorono, Darmstadt, Germany) in one orally-administered dose. For the determination of responsiveness, we used the reduction of blood Phe-after BH_4_ administration as the main criteria, while having in mind that alternative definitions of responsiveness exist [3]. A “responder” was defined when the reduction of blood Phe-level was ≥ 30% after 24 h of BH_4_ administration and a “non-responder” at smaller reductions [3,4].

### 2.4 Blood collection and amino acids measurements

**Fig. 4:**
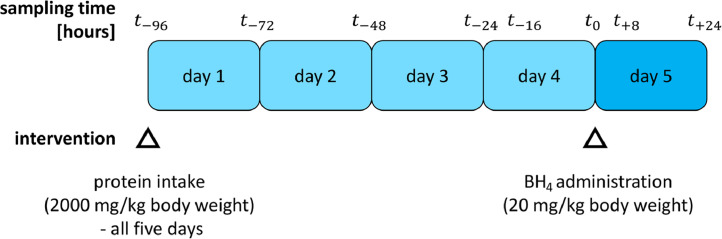
Study protocol and blood samples collection.

Blood collection was performed four days (–96 h; t_–96_), three days (–72 h; t_–72_), two days (–48 h; t_–48_), one day (–24 h; t_–24_), 16 h (t_–16_), and moments prior (t_0_) to BH_4_ administration, and 8 and 24 h (t_+8_; t_+24_) later (Fig. 4). Measurements at t_–96_, t_–72_ and t_–48_ were done only at 9 patients while all other measurements were done in all included patients. Following collection, blood was smeared on a special filter paper and dried at room temperature for approximately 3 h. Phe-and Tyr-concentrations were measured from dried blood spots by tandem mass spectrometry (Perkin Elmer PE 200 HPLC with AB Sciex 3200 QTrap).

In the first three days, blood samples were collected from only 9 patients. Day 4 (t_0_) represents the day before the administration of BH_4_. Responsiveness to BH_4_ was determined by the reduction from baseline of blood Phe, measured at t_+24_. Abbreviations: BH_4_ – tetrahydrobiopterin, Phe - phenylalanine.

### 2.5 Statistical analysis

IBM SPSS Statistics, version 26.0 (IBM, USA) was used for statistical analysis. Data was visualized in Microsoft Office 365 Excel (Microsoft Corporation, USA). The significance of Phe/Tyr-ratios change in responder and non-responder groups and in three metabolic phenotype groups was calculated with Wilcoxon matched-pairs signed-rank test. Mann-Whitney U test was used for comparison between responders and non-responders and between different phenotype groups. A *p* value of ≤ 0.05 was considered as statistically significant.

## Ethics Statements

All procedures followed were in accordance with the ethical standards of the responsible committee on human experimentation (institutional and national) and with the 2000 revision of Helsinki declaration. Written informed consent was obtained from the patients/patient's parents for inclusion into the study and for anonymized data publication and are available for review upon request. This article does not contain any studies with animal subjects performed by any of the authors.

## CRediT authorship contribution statement

**Barbka Repic Lampret:** Methodology, Investigation, Writing – original draft. **Mojca Zerjav Tansek:** Investigation, Conceptualization, Writing – original draft. **Blaz Groselj:** Investigation, Formal analysis, Writing – original draft. **Jaka Sikonja:** Investigation, Formal analysis, Visualization, Writing – original draft. **Tadej Battelino:** Investigation, Conceptualization, Supervision, Writing – review & editing. **Urh Groselj:** Investigation, Writing – review & editing, Conceptualization.

## Declaration of Competing Interest

The authors declare that they have no known competing financial interests or personal relationships that could have appeared to influence the work reported in this paper.the authors declare the following financial interests/personal relationships which may be considered as potential competing interests:
